# Supplementary material for: Distinct evolutionary trajectories of subgenomic centromeres in polyploid wheat
Source: Genome Biol. 2025 Sep 9;26:271. doi: 10.1186/s13059-025-03759-4 (PMC12418699; doi:10.1186/s13059-025-03759-4)
Supplement: Supplementary file 1 — Additional file 1. This file contains Figures S1-S12. [file 13059_2025_3759_MOESM1_ESM.pdf]

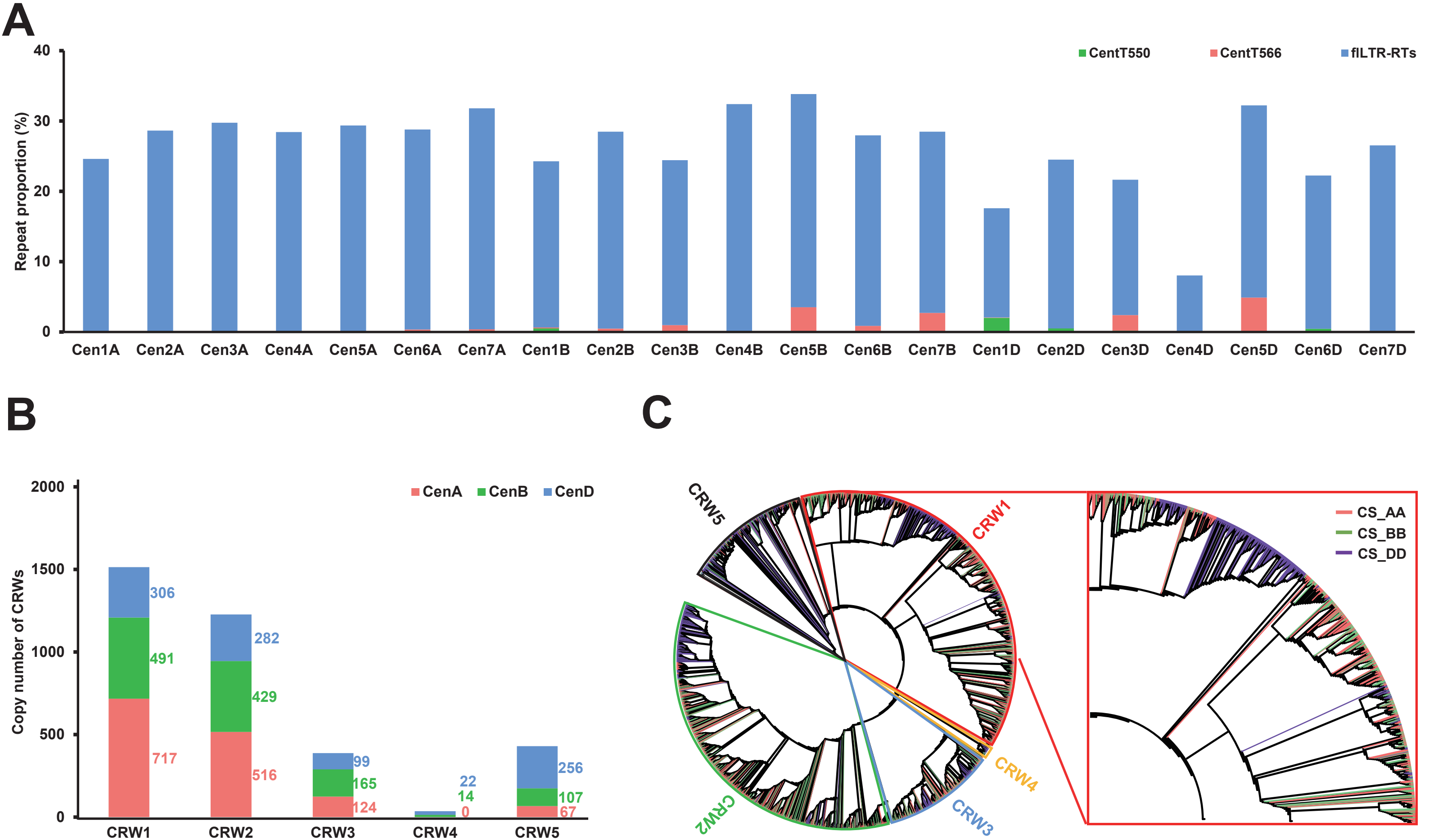
A
CentT550
CentT566
flLTR-RTs
Repeat proportion (%)
40
30
20
10
0
Cen1A
Cen2A
Cen3A
Cen4A
Cen5A
Cen6A
Cen7A
Cen1B
Cen2B
Cen3B
Cen4B
Cen5B
Cen6B
Cen7B
Cen1D
Cen2D
Cen3D
Cen4D
Cen5D
Cen6D
Cen7D
B
CenA
CenB
CenD
Copy number of CRWs
2000
1500
1000
500
0
306
491
717
282
429
516
99
165
124
22
14
0
256
107
67
CRW1
CRW2
CRW3
CRW4
CRW5
C
CRW5
CRW1
CRW2
CRW3
CRW4
CS_AA
CS_BB
CS_DD

Fig. S1. Distribution and statistics of repetitive sequences in *T. aestivum*. (A) Proportions of flLTR-RTs and satellite sequences in the 21 wheat chromosomes. (B) Copy numbers of CRWs in the AA, BB, and DD subcentromeres of *T. aestivum*. The numbers next to the bars indicate the copy counts. (C) Phylogenetic tree illustrating the interweaving of CRWs between subgenomes AA and BB, with branch lengths ignored. CRWs from AA subcentromeres are shown in pink, CRWs from BB subcentromeres in green, and CRWs from DD subcentromeres in purple. The right inset provides an enlarged view of the distribution of phylogenetic branches in CRW1.

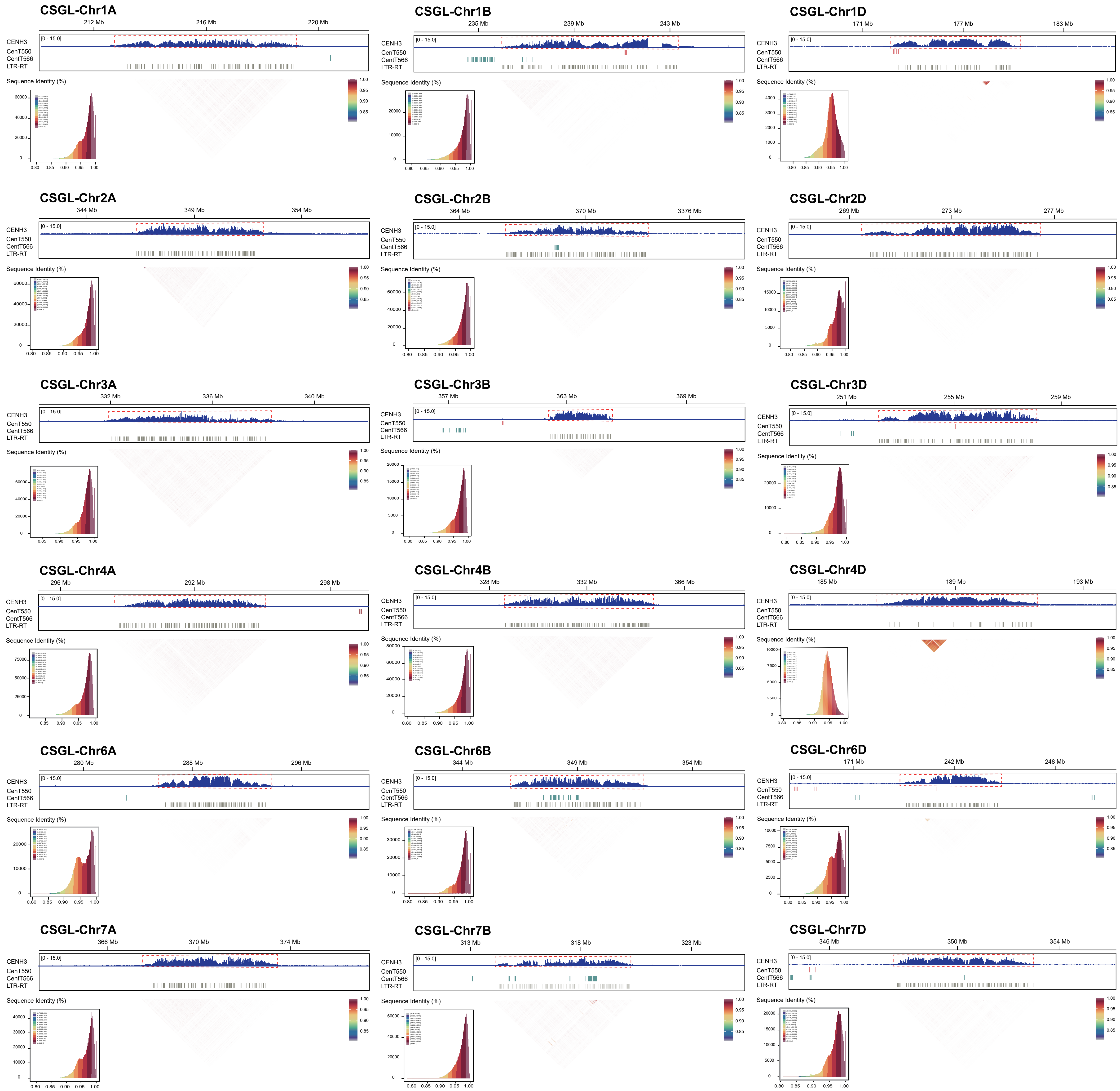

CSGL-Chr1A
212 Mb
216 Mb
220 Mb
CENH3
[0 - 15.0]
CenT550
CentT566
LTR-RT
Sequence Identity (%)
CSGL-Chr1B
235 Mb
239 Mb
243 Mb
CSGL-Chr1D
171 Mb
177 Mb
183 Mb
CSGL-Chr2A
344 Mb
349 Mb
354 Mb
CSGL-Chr2B
364 Mb
370 Mb
3376 Mb
CSGL-Chr2D
269 Mb
273 Mb
277 Mb
CSGL-Chr3A
332 Mb
336 Mb
340 Mb
CSGL-Chr3B
357 Mb
363 Mb
369 Mb
CSGL-Chr3D
251 Mb
255 Mb
259 Mb
CSGL-Chr4A
296 Mb
292 Mb
298 Mb
CSGL-Chr4B
328 Mb
332 Mb
366 Mb
CSGL-Chr4D
185 Mb
189 Mb
193 Mb
CSGL-Chr6A
280 Mb
288 Mb
296 Mb
CSGL-Chr6B
344 Mb
349 Mb
354 Mb
CSGL-Chr6D
171 Mb
242 Mb
248 Mb
CSGL-Chr7A
366 Mb
370 Mb
374 Mb
CSGL-Chr7B
313 Mb
318 Mb
323 Mb
CSGL-Chr7D
346 Mb
350 Mb
354 Mb

Fig. S2. Fine structure of centromeric repeat arrays in hexaploid wheat *T. aestivum*. Characteristics of the 18 centromeres (5th homoeologous group in Fig. 1A) in the CS accession. Different layers display the CENH3 enrichment [$\log_2$(ChIP/Input)], the distribution of centromeric satellites centT550 and CentT566, annotations of the centromeric flLTR-RTs, and a heatmap of pairwise satellite sequence similarity.

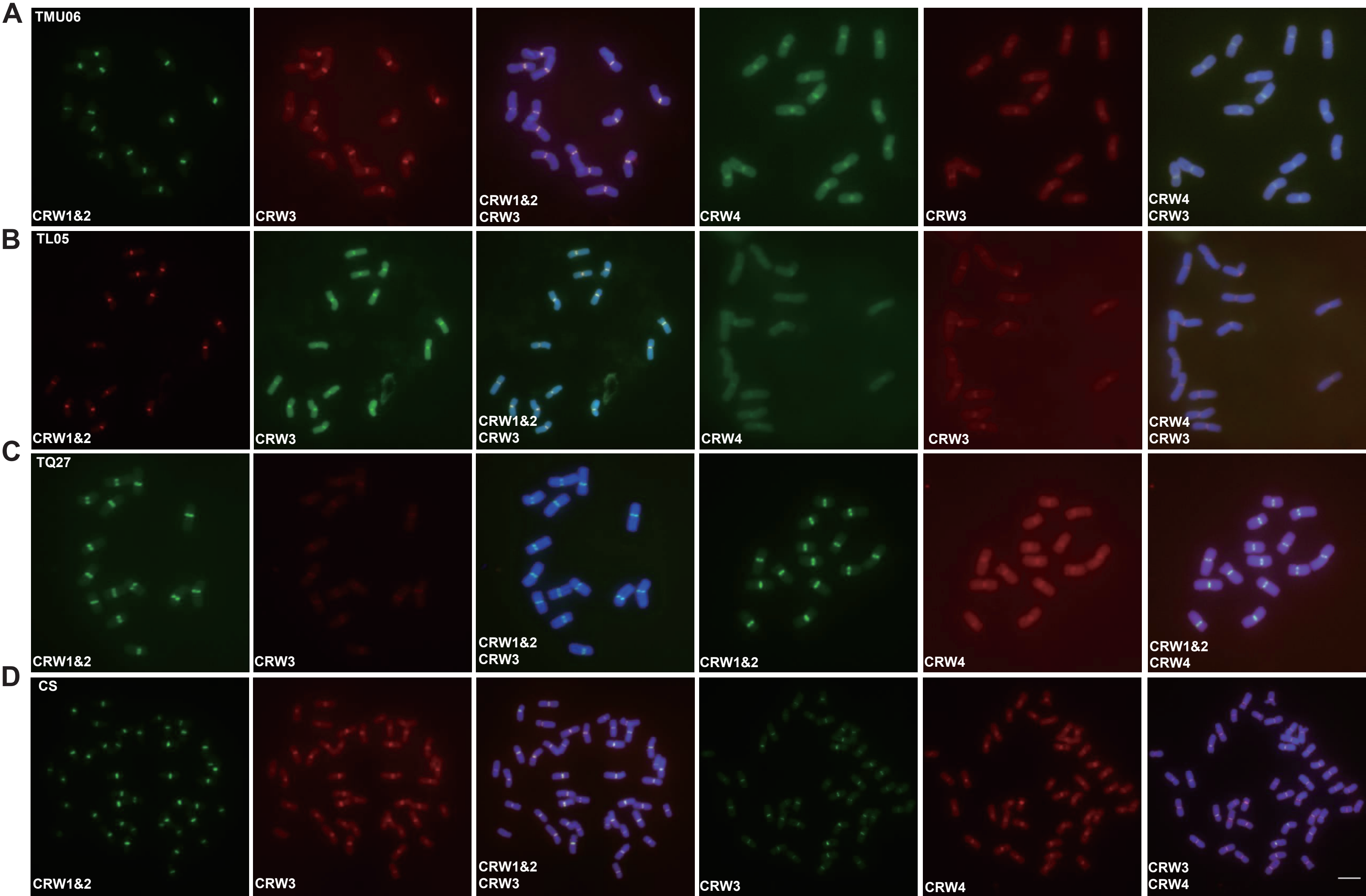
A
TMU06
CRW1&2
CRW3
CRW1&2
CRW3
CRW4
CRW3
CRW4
CRW3
B
TL05
CRW1&2
CRW3
CRW1&2
CRW3
CRW4
CRW3
CRW4
CRW3
C
TQ27
CRW1&2
CRW3
CRW1&2
CRW3
CRW1&2
CRW4
CRW1&2
CRW4
D
CS
CRW1&2
CRW3
CRW1&2
CRW3
CRW3
CRW4
CRW3
CRW4

Fig. S3. The centromere location of CRWs in different wheat Lines. The centromeric region is mainly enriched with CRW1&2, with trace amounts of CRW4 and CRW3. (A) FISH signals of CRWs in the wheat A genome donor *T. urartu* TMU06. (B) FISH signals of CRWs in *Ae. longissima* TL05. (C) FISH signals of CRWs in the wheat D genome donor *Ae. tauschii* TQ27. (D) FISH signals of CRWs in *T. aestivum* CS. For FISH results, blue indicates chromosomes counterstained with 4',6-diamidino-2-phenylindole, the CRW1&2 are labeled in green, and CRW3 and CRW4 are labeled in red or green, depending on the plot subscript. Scale bar = 10 μm.

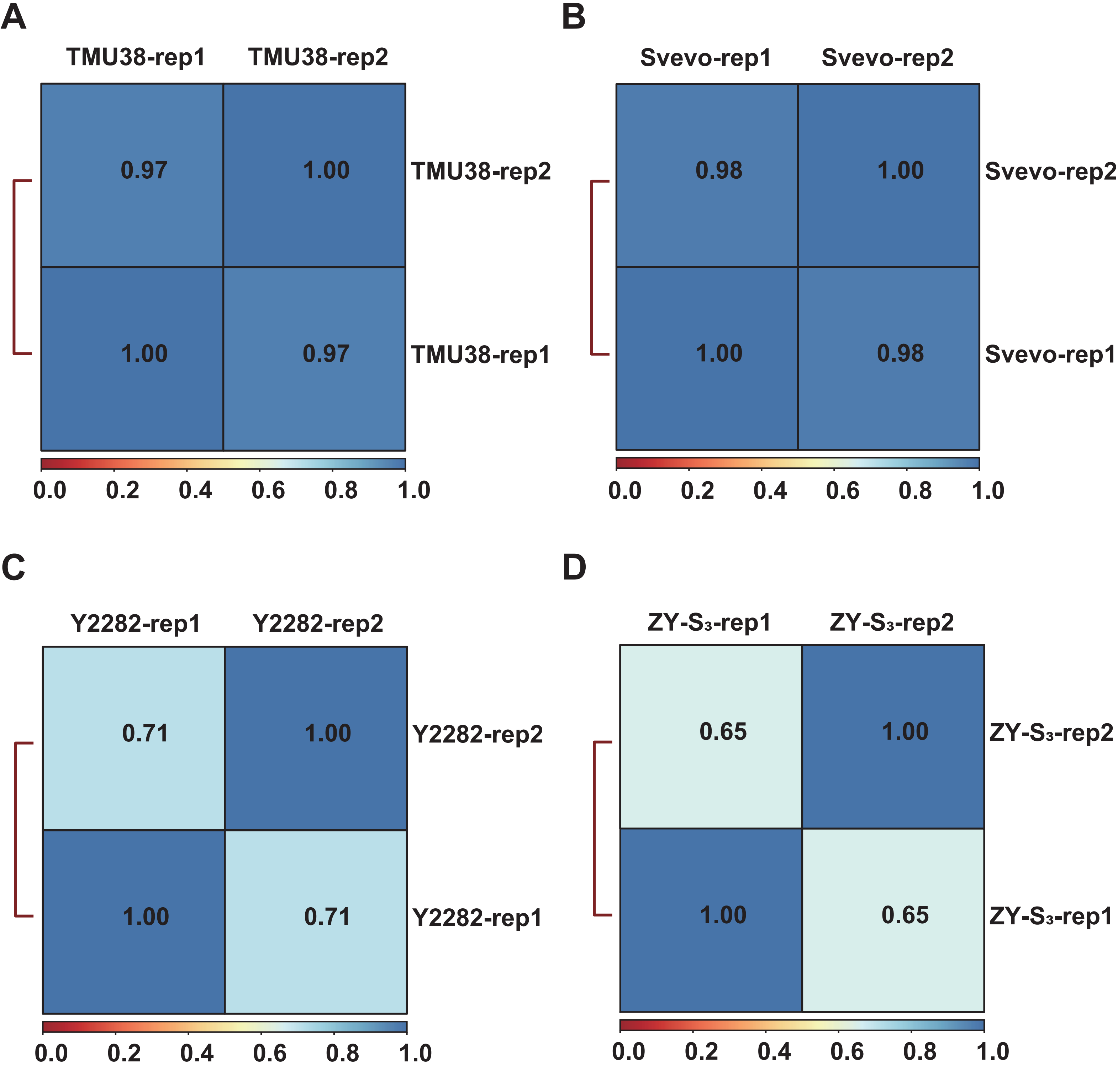

A
TMU38-rep1
TMU38-rep2
0.97
1.00
TMU38-rep2
1.00
0.97
TMU38-rep1
0.0
0.2
0.4
0.6
0.8
1.0
B
Svevo-rep1
Svevo-rep2
0.98
1.00
Svevo-rep2
1.00
0.98
Svevo-rep1
0.0
0.2
0.4
0.6
0.8
1.0
C
Y2282-rep1
Y2282-rep2
0.71
1.00
Y2282-rep2
1.00
0.71
Y2282-rep1
0.0
0.2
0.4
0.6
0.8
1.0
D
ZY-S3-rep1
ZY-S3-rep2
0.65
1.00
ZY-S3-rep2
1.00
0.65
ZY-S3-rep1
0.0
0.2
0.4
0.6
0.8
1.0

Fig. S4. Basic information on the correlation between ChIP-seq replicates. Spearman's correlation of read density (with a bin size of 10 kb) was calculated between technical replicates using the deepTools software (v3.5.0). *T. urartu* (A^uA^u, TMU38) (A), *T. turgidum* (BBAA, Svevo) (B), *Ae. tauschii* (DD, Y2282) (C), the third-generation hybrid offspring *T. turgidum* ssp. *dicoccoides* × *Ae. tauschii* (BBAADD, Za-Y2 S_3) (D).

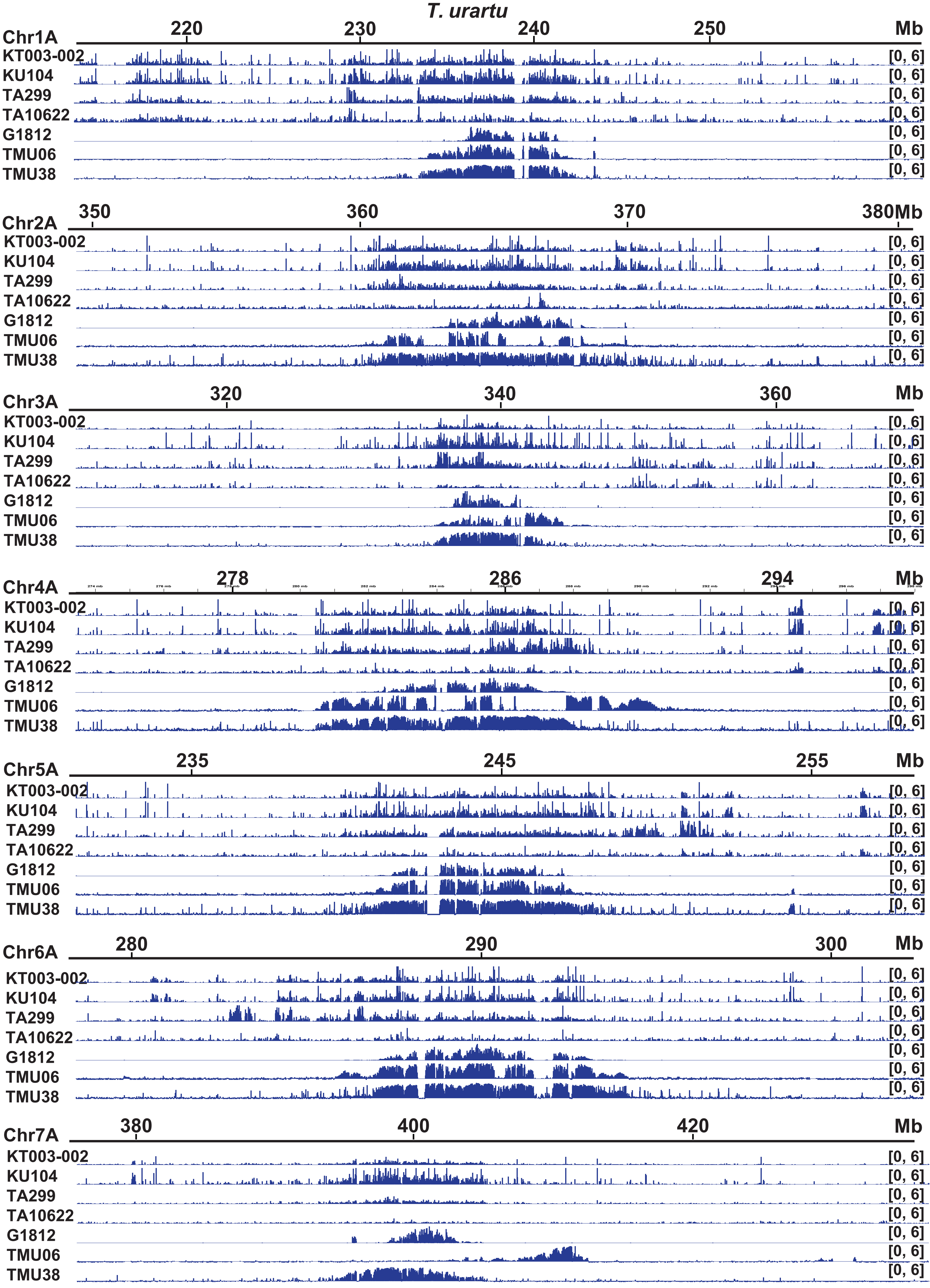

T. urartu
Chr1A
220
230
240
250
Mb
KT003-002
KU104
TA299
TA10622
G1812
TMU06
TMU38
[0, 6]
Chr2A
350
360
370
380Mb
Chr3A
320
340
360
Mb
Chr4A
278
286
294
Mb
Chr5A
235
245
255
Mb
Chr6A
280
290
300
Mb
Chr7A
380
400
420
Mb

Fig. S5. Genome-wide mapping of CENH3 ChIP-seq reads from four *T. monococcum* accessions (KT003-002, KU104, TA299, and TA10622) and three different *T. urartu* accessions (G1812, TMU06, and TMU38) to the *T. urartu* reference genome. Distribution of the density of seven CENH3 enrichment [$\log_2$(ChIP/Input)] samples along the chromosomes of *T. urartu*. Only continuous CENH3 enrichment regions can be detected in all three *T. urartu* accessions.

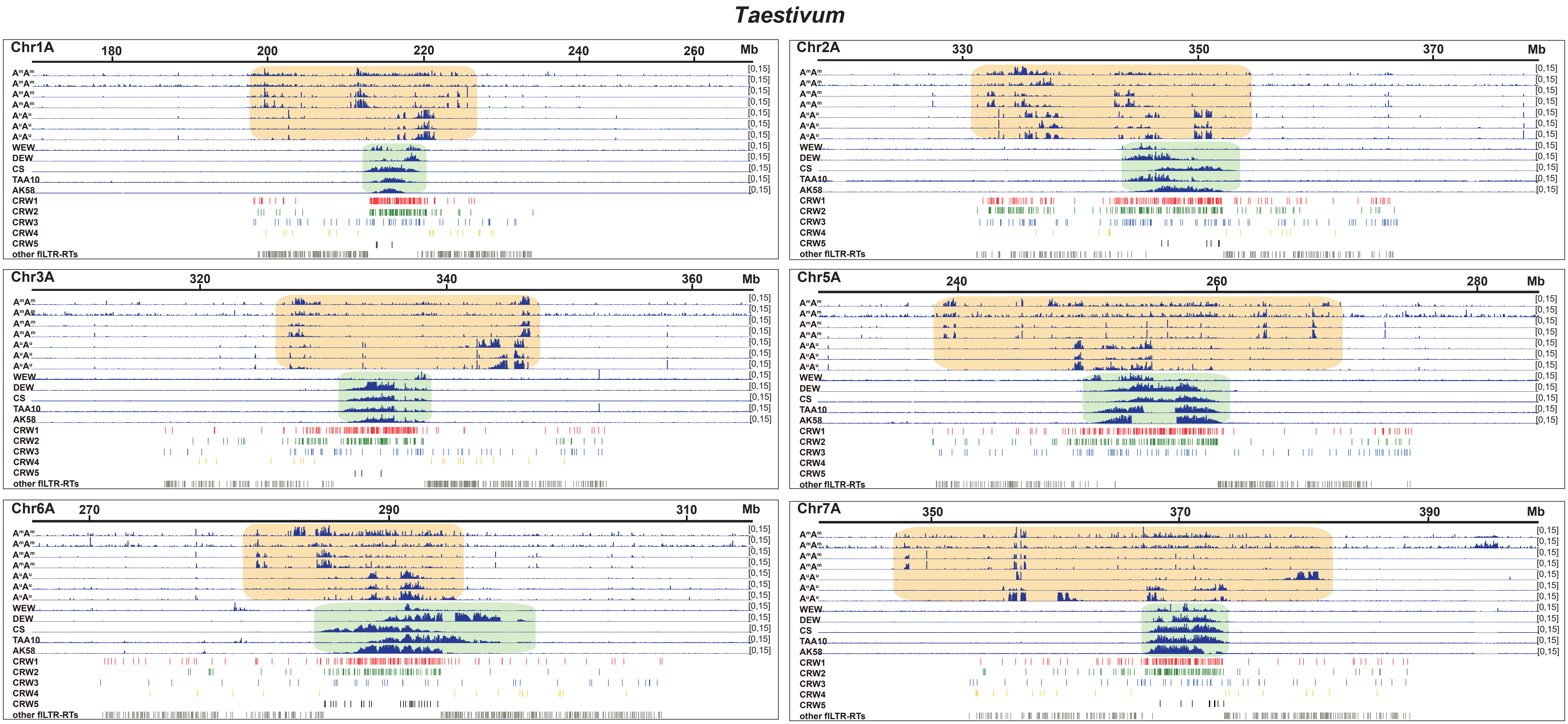
Taestivum
Chr1A
180
200
220
240
260
Mb
Chr2A
330
350
370
Mb
Chr3A
320
340
360
Mb
Chr5A
240
260
280
Mb
Chr6A
270
290
310
Mb
Chr7A
350
370
390
Mb
AᵐAᵐ
AᵘAᵘ
WEW
DEW
CS
TAA10
AK58
CRW1
CRW2
CRW3
CRW4
CRW5
other flLTR-RTs
[0,15]

Fig. S6. Diploid wheat exhibits a sporadic distribution pattern along the *T. aestivum* AA pericentromeres. The density distribution of CENH3 [$\log_2$(ChIP/Input)] along the chromosomes of the AA subgenome of hexaploid wheat *T. aestivum*. The first four tracks (1-4) are *T. monococcum* (A^mA^m, accessions TA299, TA10622, KT003-002, and KU104). The next three (5-7) are *T. urartu* (A^uA^u, accessions TMU06, G1812, and TMU38). Tracks eight and nine (8-9) are wild emmer wheat (WEW, accession Zavitan) and domesticated emmer wheat (DEW, accession Svevo), belonging to *T. turgidum* (BBAA). Tracks ten to twelve (10-12) are *T. aestivum* (accessions CS, TAA10, and Aikang58 (AK58)). The distribution of CRW1 (red), CRW2 (green), CRW3 (blue), CRW4 (yellow), CRW5 (black), and other flLTR-RTs (gray) in pericentromeres is shown in the respective tracks.

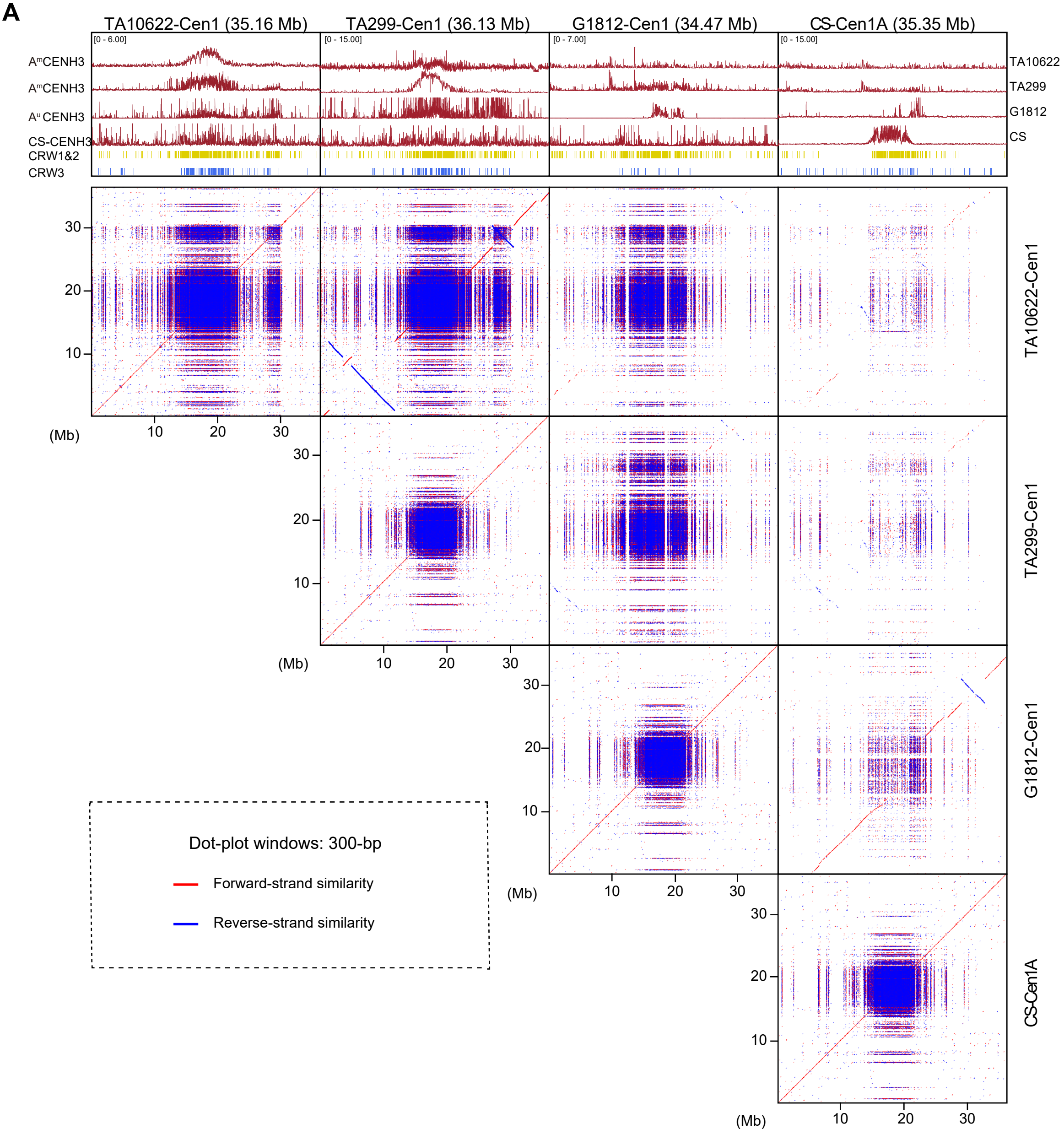
A
TA10622-Cen1 (35.16 Mb)
TA299-Cen1 (36.13 Mb)
G1812-Cen1 (34.47 Mb)
CS-Cen1A (35.35 Mb)
[0 - 6.00]
[0 - 15.00]
[0 - 7.00]
[0 - 15.00]
AᵐCENH3
AᵐCENH3
Aᵘ CENH3
CS-CENH3
CRW1&2
CRW3
TA10622
TA299
G1812
CS
TA10622-Cen1
TA299-Cen1
G1812-Cen1
CS-Cen1A
(Mb)
10
20
30
Dot-plot windows: 300-bp
Forward-strand similarity
Reverse-strand similarity

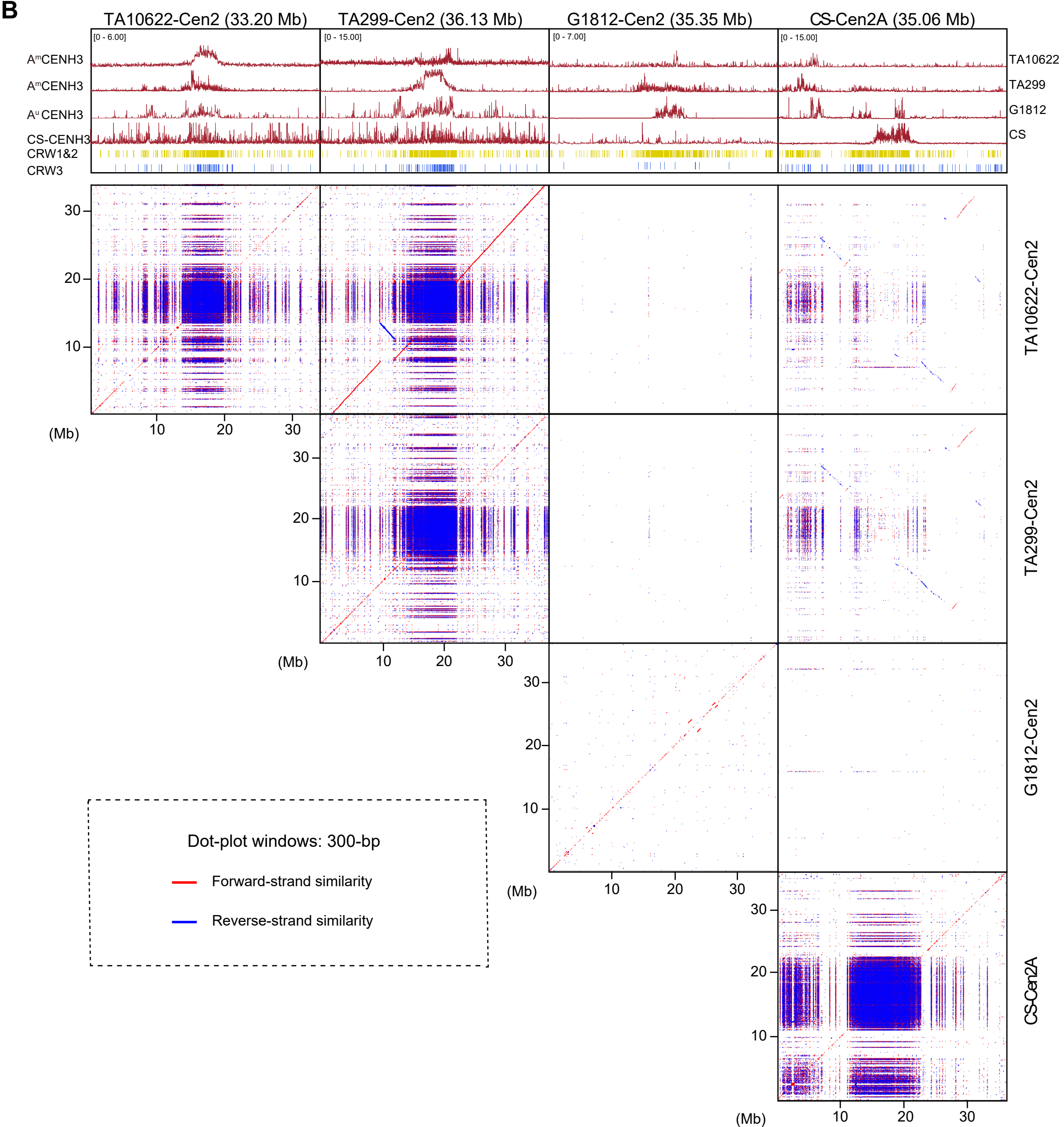

B
TA10622-Cen2 (33.20 Mb)
TA299-Cen2 (36.13 Mb)
G1812-Cen2 (35.35 Mb)
CS-Cen2A (35.06 Mb)
[0 - 6.00]
[0 - 15.00]
[0 - 7.00]
[0 - 15.00]
A^mCENH3
A^mCENH3
A^u CENH3
CS-CENH3
CRW1&2
CRW3
TA10622
TA299
G1812
CS
TA10622-Cen2
TA299-Cen2
G1812-Cen2
CS-Cen2A
(Mb)
Dot-plot windows: 300-bp
Forward-strand similarity
Reverse-strand similarity

C

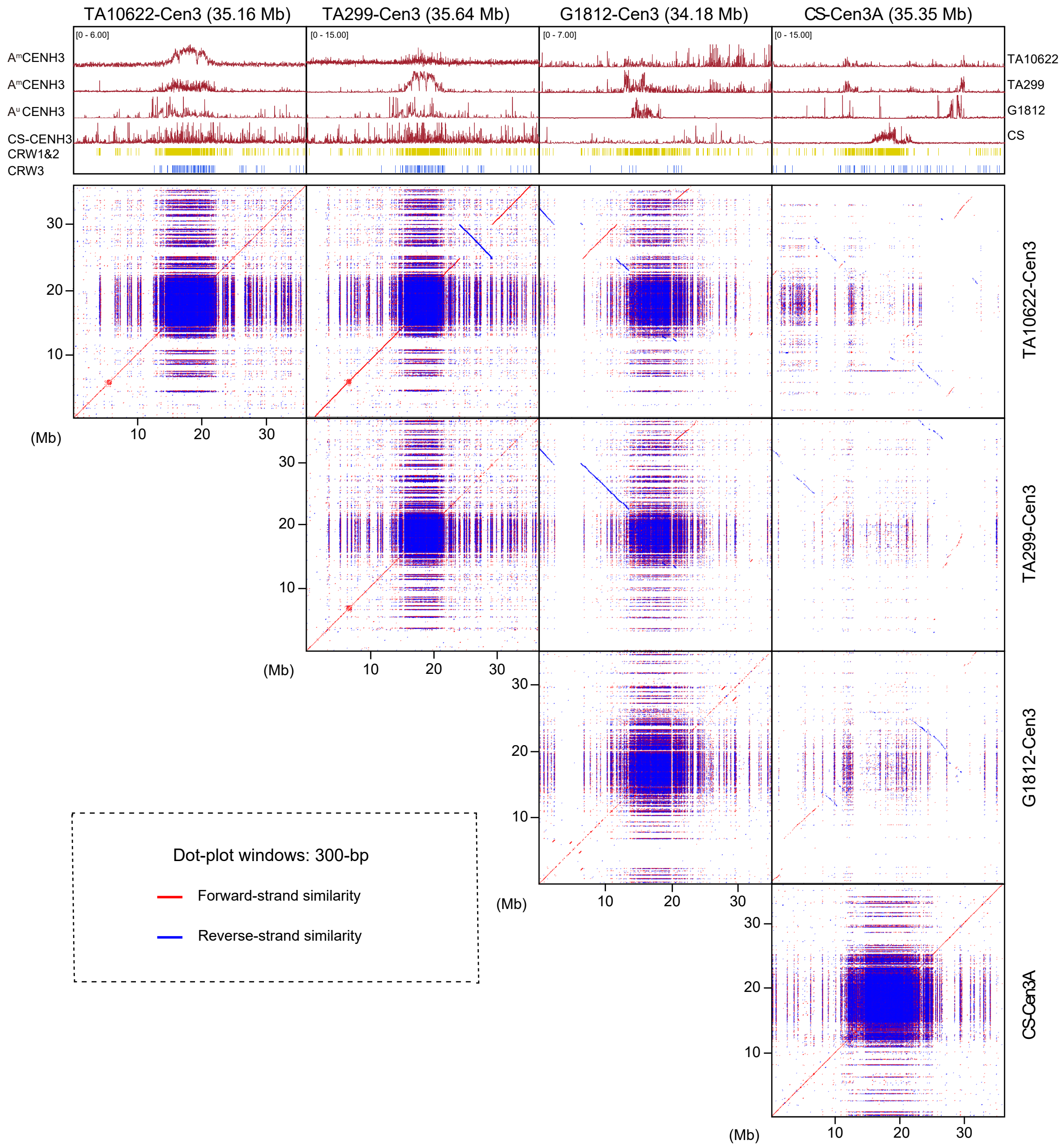
TA10622-Cen3 (35.16 Mb)
TA299-Cen3 (35.64 Mb)
G1812-Cen3 (34.18 Mb)
CS-Cen3A (35.35 Mb)
[0 - 6.00]
[0 - 15.00]
[0 - 7.00]
[0 - 15.00]
A^m CENH3
A^m CENH3
A^u CENH3
CS-CENH3
CRW1&2
CRW3
TA10622
TA299
G1812
CS
TA10622-Cen3
TA299-Cen3
G1812-Cen3
CS-Cen3A
(Mb)
10
20
30
Dot-plot windows: 300-bp
Forward-strand similarity
Reverse-strand similarity

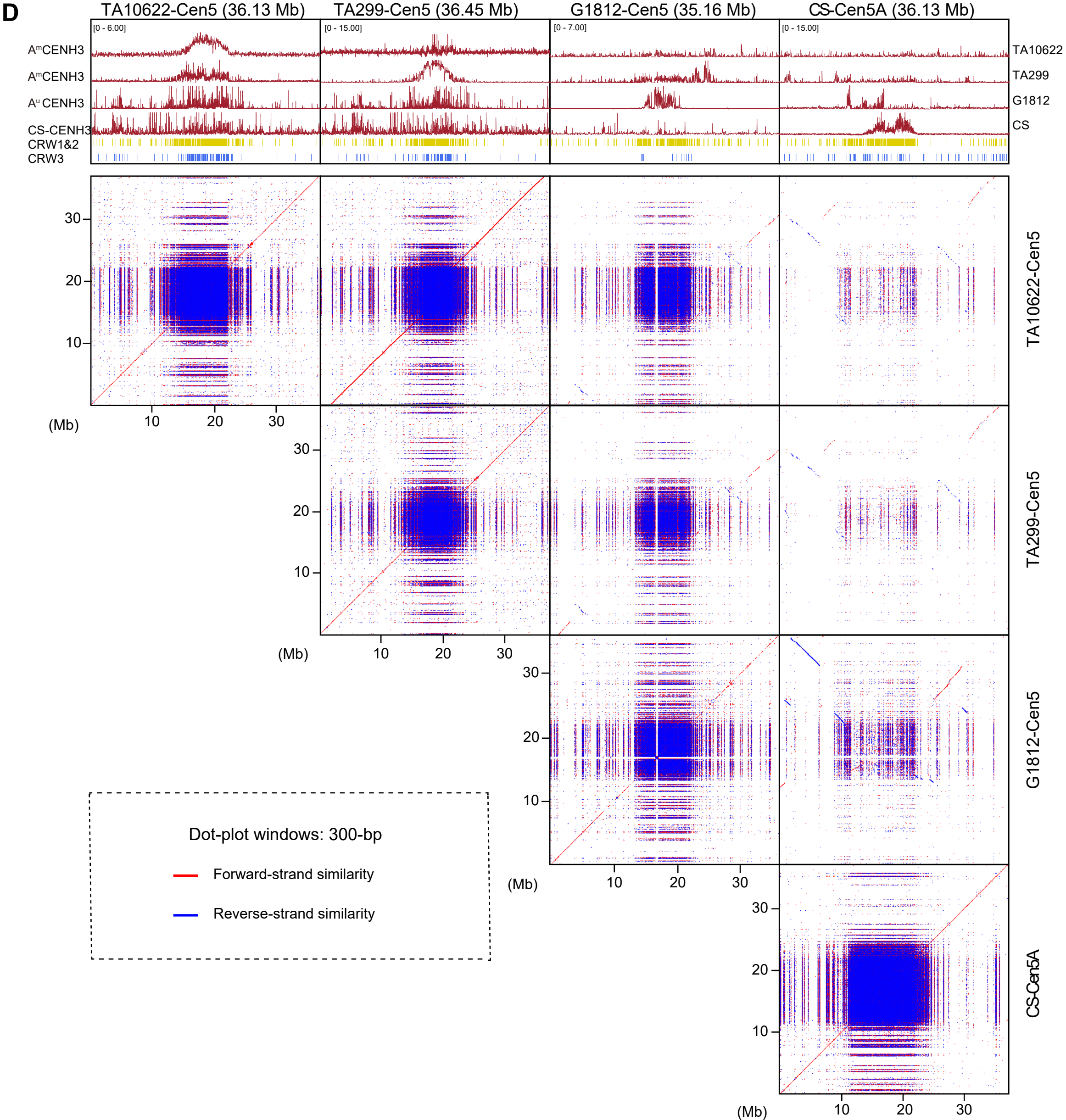

D
TA10622-Cen5 (36.13 Mb)
TA299-Cen5 (36.45 Mb)
G1812-Cen5 (35.16 Mb)
CS-Cen5A (36.13 Mb)
[0 - 6.00]
[0 - 15.00]
[0 - 7.00]
[0 - 15.00]
AmCENH3
AmCENH3
Au CENH3
CS-CENH3
CRW1&2
CRW3
TA10622
TA299
G1812
CS
TA10622-Cen5
TA299-Cen5
G1812-Cen5
CS-Cen5A
(Mb)
10
20
30
Dot-plot windows: 300-bp
Forward-strand similarity
Reverse-strand similarity

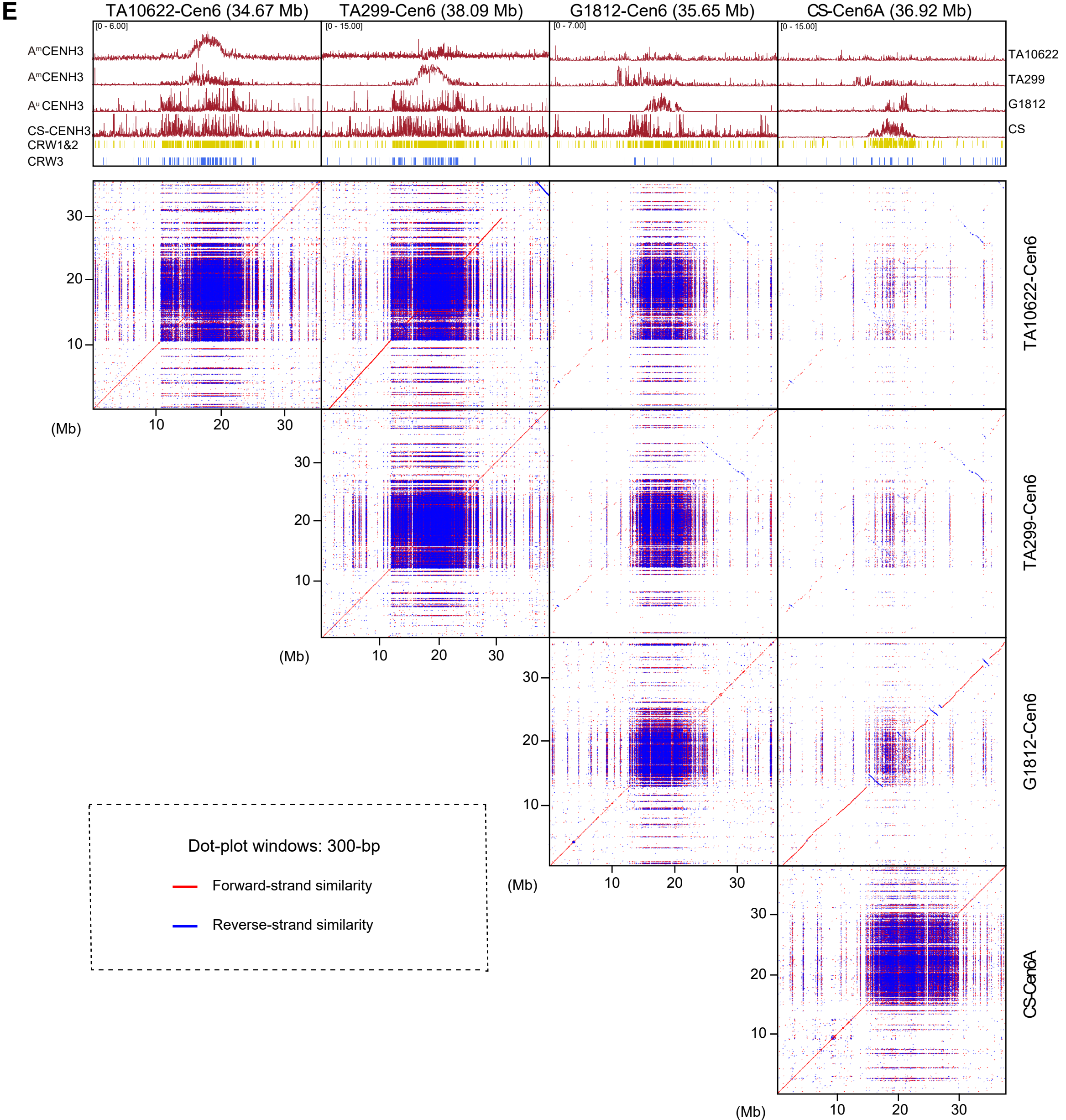
E
TA10622-Cen6 (34.67 Mb)
TA299-Cen6 (38.09 Mb)
G1812-Cen6 (35.65 Mb)
CS-Cen6A (36.92 Mb)
[0 - 6.00]
[0 - 15.00]
[0 - 7.00]
[0 - 15.00]
A^m CENH3
A^m CENH3
A^u CENH3
CS-CENH3
CRW1&2
CRW3
TA10622
TA299
G1812
CS
TA10622-Cen6
TA299-Cen6
G1812-Cen6
CS-Cen6A
(Mb)
10
20
30
Dot-plot windows: 300-bp
Forward-strand similarity
Reverse-strand similarity

F

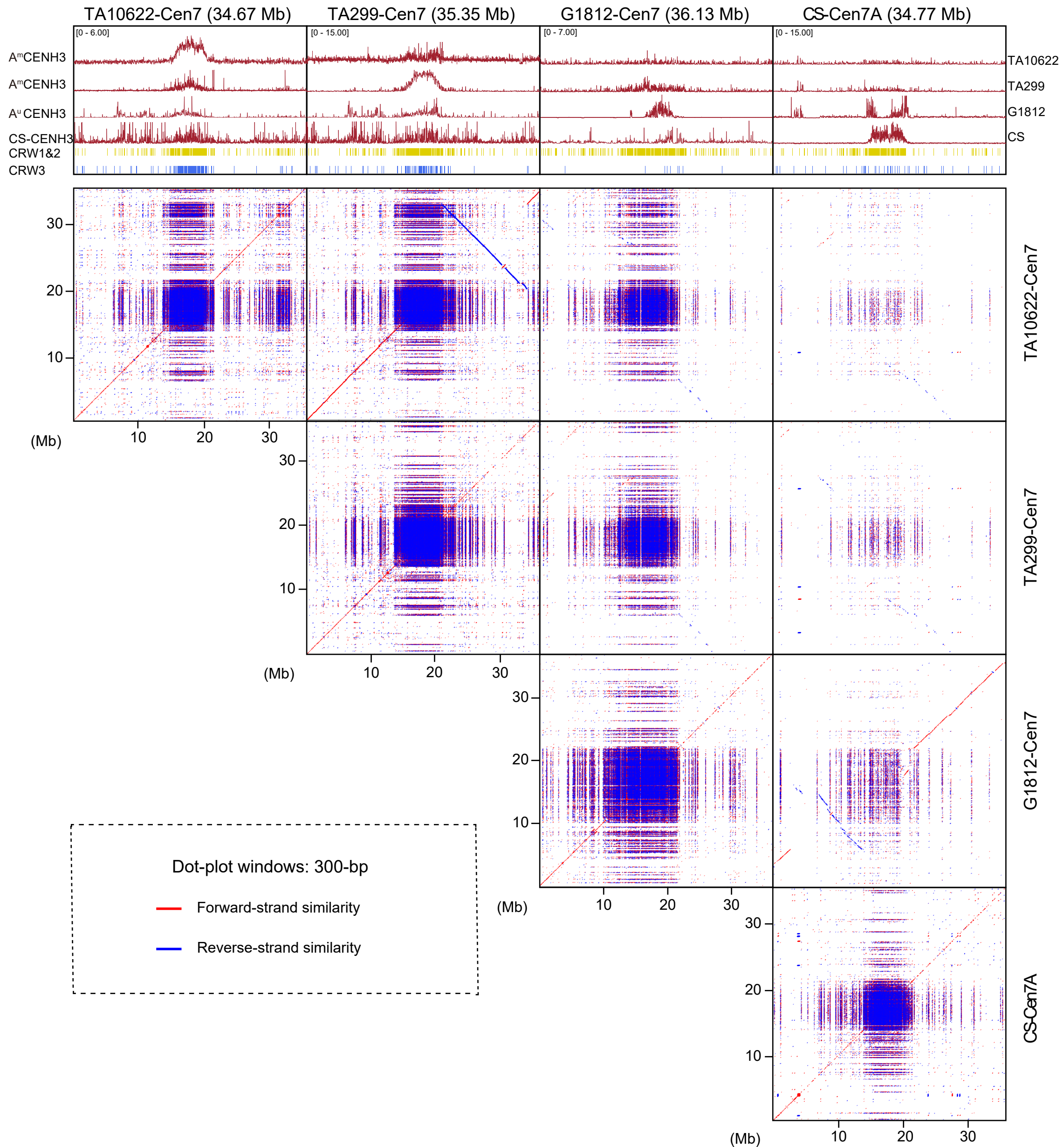

TA10622-Cen7 (34.67 Mb)
TA299-Cen7 (35.35 Mb)
G1812-Cen7 (36.13 Mb)
CS-Cen7A (34.77 Mb)
[0 - 6.00]
[0 - 15.00]
[0 - 7.00]
[0 - 15.00]
A^m CENH3
A^m CENH3
A^u CENH3
CS-CENH3
CRW1&2
CRW3
TA10622
TA299
G1812
CS
TA10622-Cen7
TA299-Cen7
G1812-Cen7
CS-Cen7A
(Mb)
10
20
30
Dot-plot windows: 300-bp
Forward-strand similarity
Reverse-strand similarity

Fig. S7. Comparative analysis of A-genome centromere architecture across wheat species. Note that there is little similarity between *T. aestivum*-Cen2A and *T. urartu*-Cen2A. (A-F) Dot plot alignments (300-bp window) of peri/centromeric regions for chromosomes 1A–7A (except 4A), comparing *T. monococcum* (domesticated einkorn TA10622, wild einkorn TA299), *T. urartu* (G1812), and *T. aestivum* (CS). Genomic coordinates (top) and CENH3 ChIP-seq coverage profiles (middle) demarcate centromere boundaries. CRWs distributions are annotated as colored bars. Forward- and reverse-strand sequence similarities are indicated in red and blue, respectively.

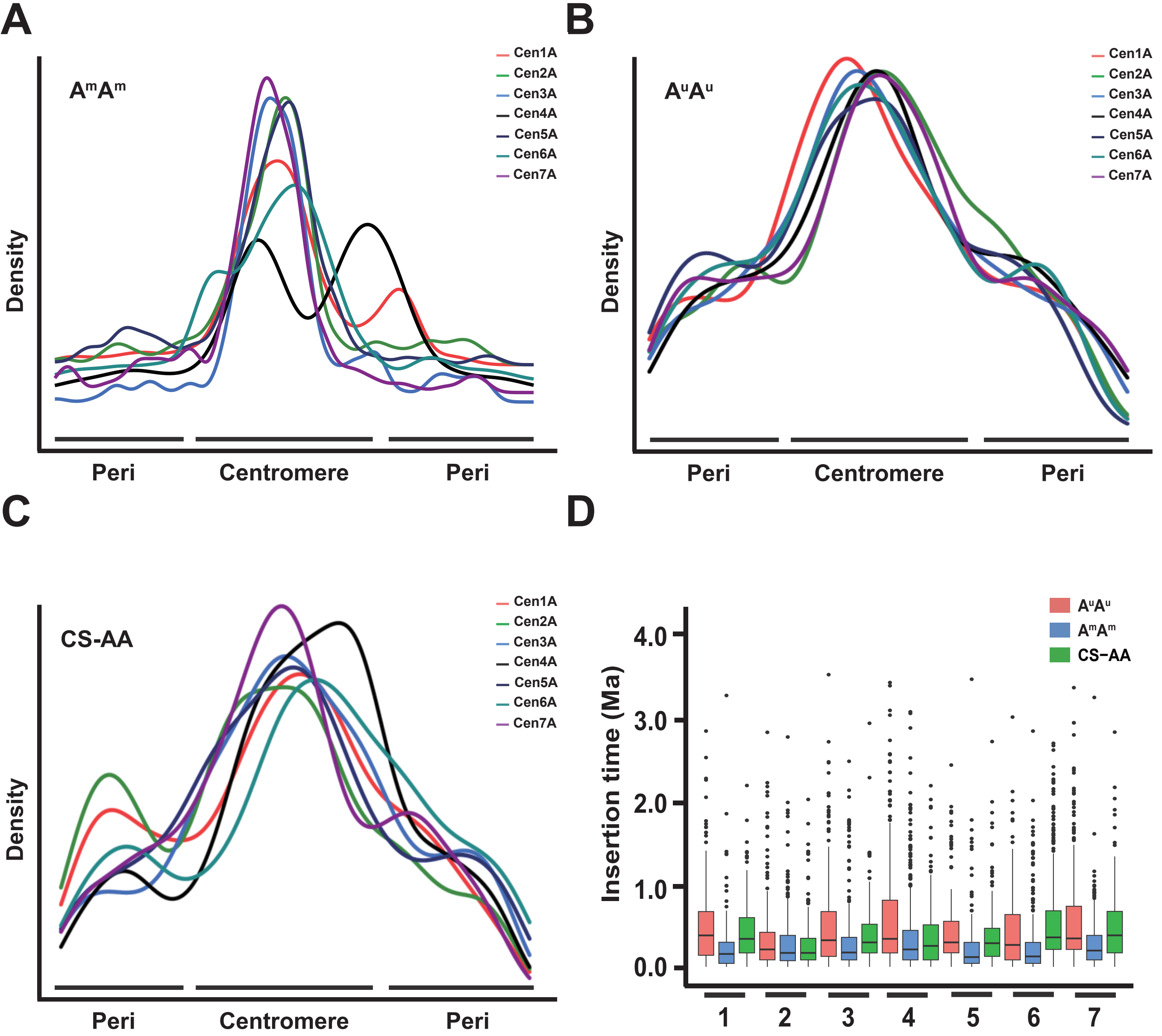

A
AᵐAᵐ
Cen1A
Cen2A
Cen3A
Cen4A
Cen5A
Cen6A
Cen7A
Density
Peri
Centromere
Peri
B
AᵘAᵘ
Cen1A
Cen2A
Cen3A
Cen4A
Cen5A
Cen6A
Cen7A
Density
Peri
Centromere
Peri
C
CS-AA
Cen1A
Cen2A
Cen3A
Cen4A
Cen5A
Cen6A
Cen7A
Density
Peri
Centromere
Peri
D
AᵘAᵘ
AᵐAᵐ
CS−AA
Insertion time (Ma)
4.0
3.0
2.0
1.0
0.0
1
2
3
4
5
6
7

Fig. S8. Evolutionary analysis of diploid and hexaploid CenA. (A-C) Kernel density estimate plot of flLTR-RTs in the AA peri/centromeres of *T. monococcum* (A), *T. urartu* (B) and AA pericentromeres of *T. aestivum* (C) across homoeologous groups Chr1 to Chr7. Peri/centromere 1A (Cen1A) is shown in red, Cen2A is green, Cen3A is blue, Cen4A is black, Cen5A is navy blue, Cen6A is cyan, and Cen7A is purple. (D) Comparison of CRWs insertion time in *T. urartu* (red), *T. monococcum* (blue), and *T. aestivum* (cyan) among homoeologous groups Chr1 to Chr7.

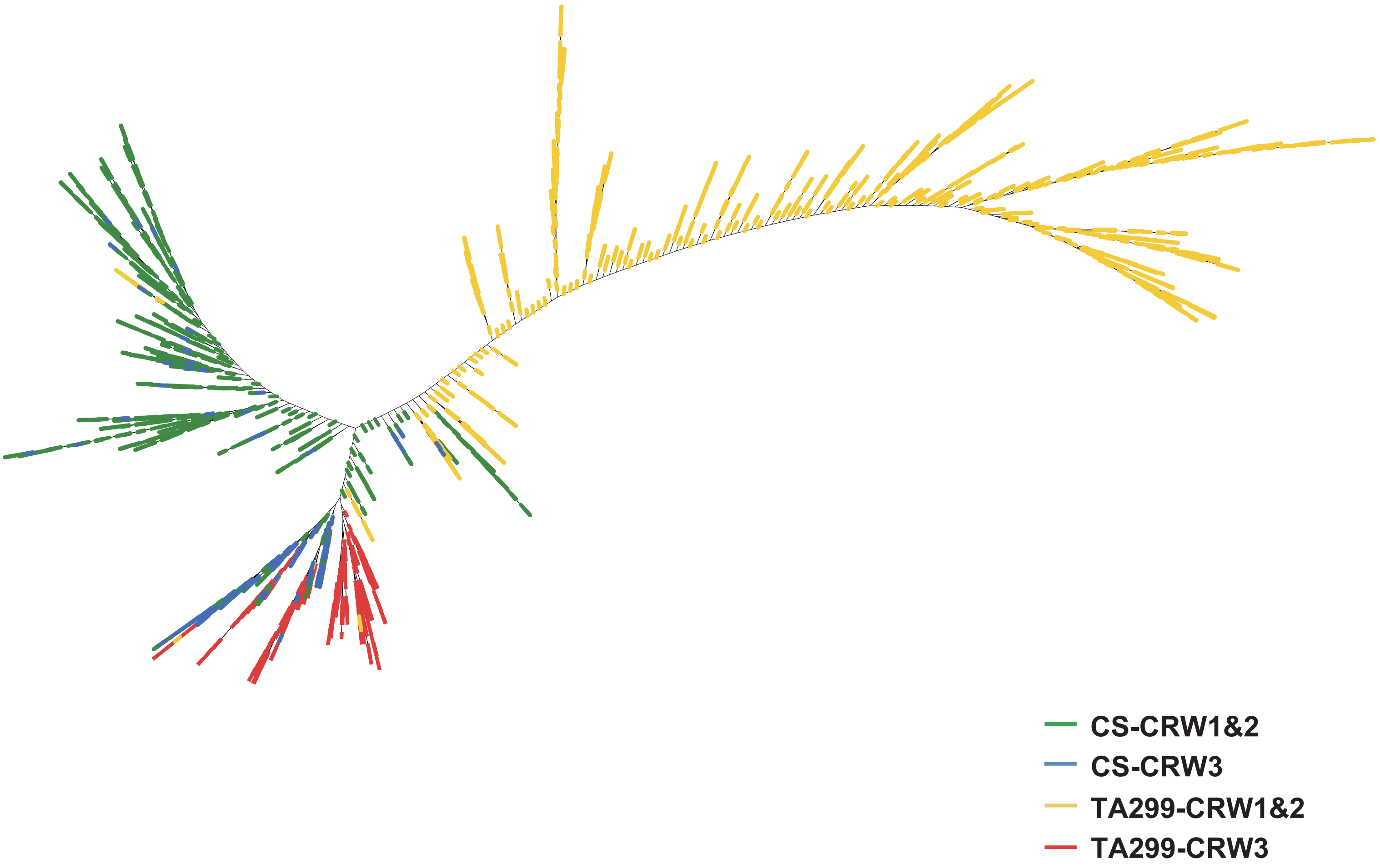
CS-CRW1&2
CS-CRW3
TA299-CRW1&2
TA299-CRW3

Fig. S9. Phylogenetic analysis of CRWs from centromeric regions of *T. monococcum* (TA299) and *T. aestivum* (CS-AA). CRWs are color-coded by subfamily. CRW1&2 (green) and CRW3 (blue) from *T. aestivum*; CRW1&2/*Cereba* (yellow) and CRW3/*Quinta* (red) from *T. monococcum*. CRW4 was excluded from analysis due to low abundance. Phylogenetic analysis demonstrates that CRW3 elements maintain conserved clusters across ploidy levels, while CRW1&2 exhibit divergent evolutionary paths between the two wheat species, suggesting distinct evolutionary dynamics among centromeric retrotransposon lineages.

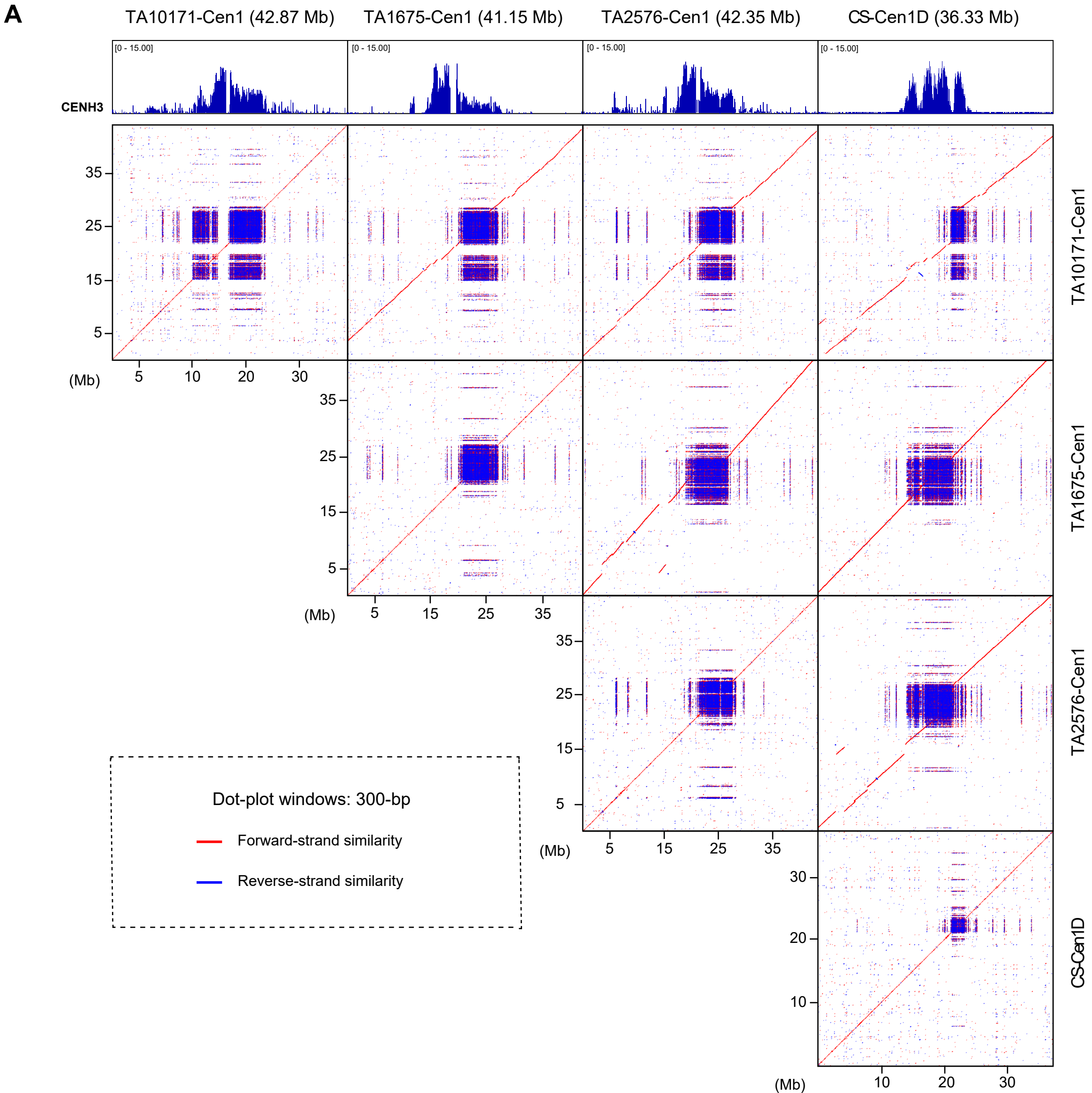

A
TA10171-Cen1 (42.87 Mb)
TA1675-Cen1 (41.15 Mb)
TA2576-Cen1 (42.35 Mb)
CS-Cen1D (36.33 Mb)
[0 - 15.00]
CENH3
TA10171-Cen1
TA1675-Cen1
TA2576-Cen1
CS-Cen1D
(Mb)
Dot-plot windows: 300-bp
Forward-strand similarity
Reverse-strand similarity

B

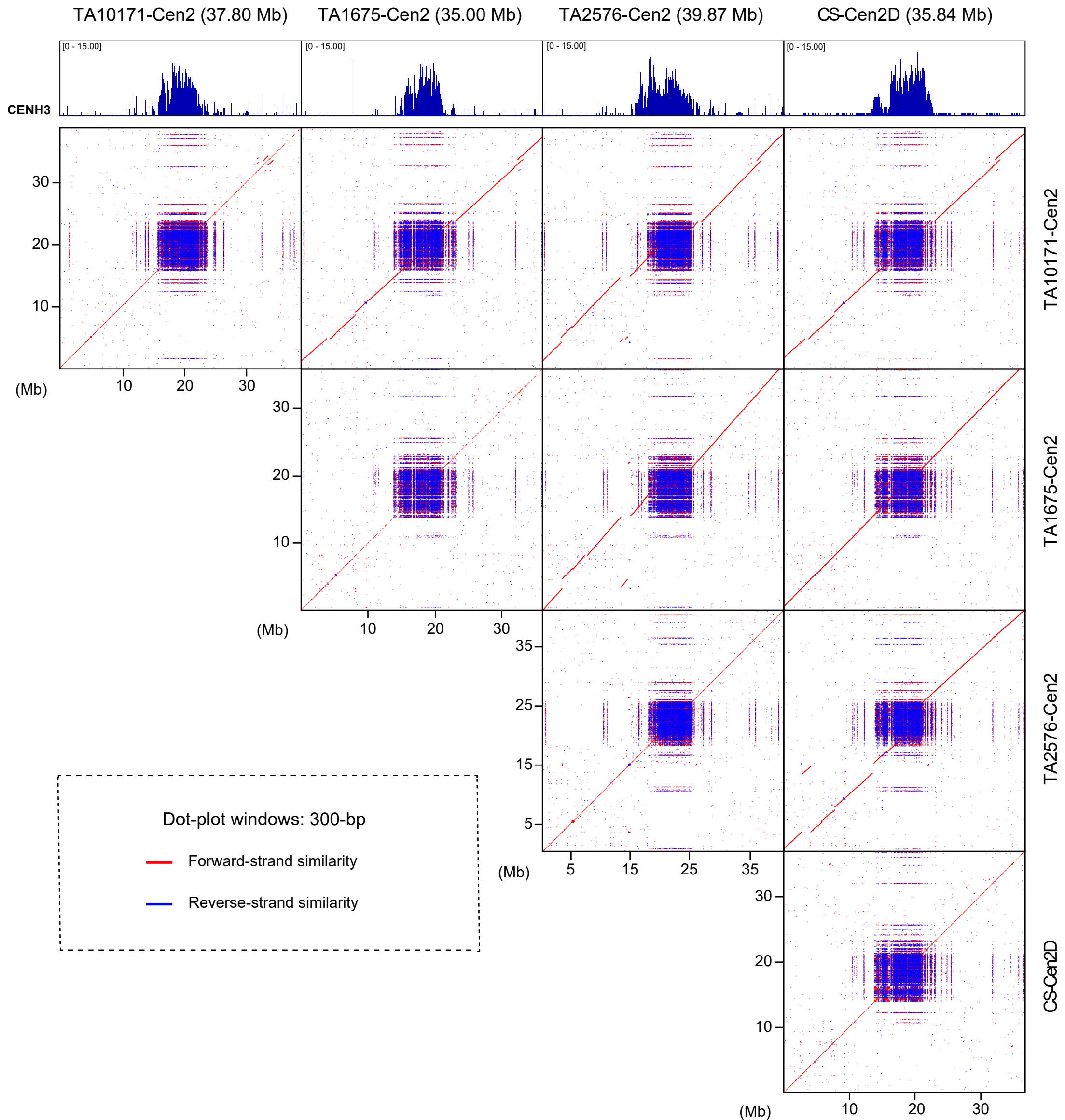
TA10171-Cen2 (37.80 Mb)
TA1675-Cen2 (35.00 Mb)
TA2576-Cen2 (39.87 Mb)
CS-Cen2D (35.84 Mb)
[0 - 15.00]
CENH3
TA10171-Cen2
TA1675-Cen2
TA2576-Cen2
CS-Cen2D
(Mb)
Dot-plot windows: 300-bp
Forward-strand similarity
Reverse-strand similarity

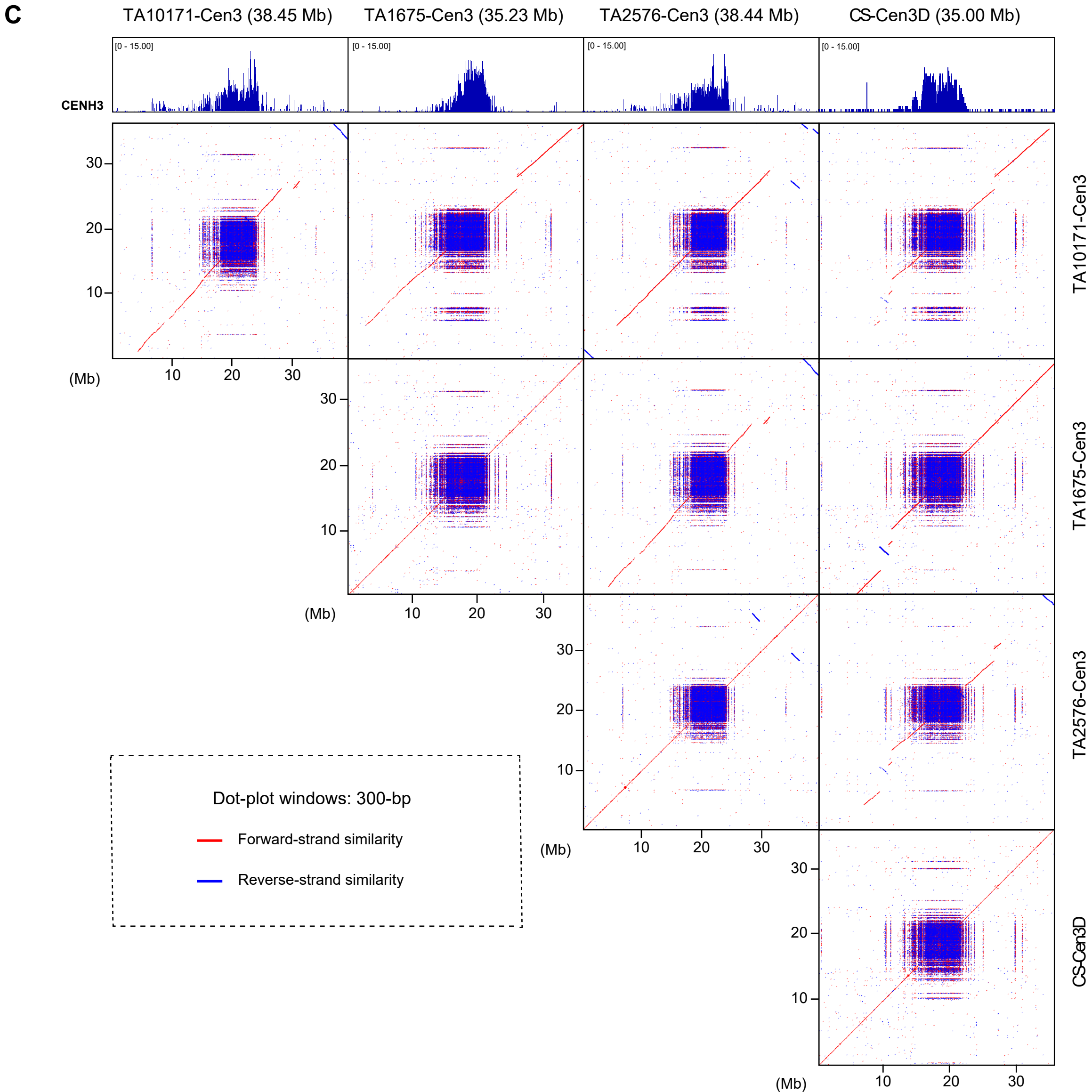
C
TA10171-Cen3 (38.45 Mb)
TA1675-Cen3 (35.23 Mb)
TA2576-Cen3 (38.44 Mb)
CS-Cen3D (35.00 Mb)
[0 - 15.00]
CENH3
TA10171-Cen3
TA1675-Cen3
TA2576-Cen3
CS-Cen3D
(Mb)
10
20
30
Dot-plot windows: 300-bp
Forward-strand similarity
Reverse-strand similarity

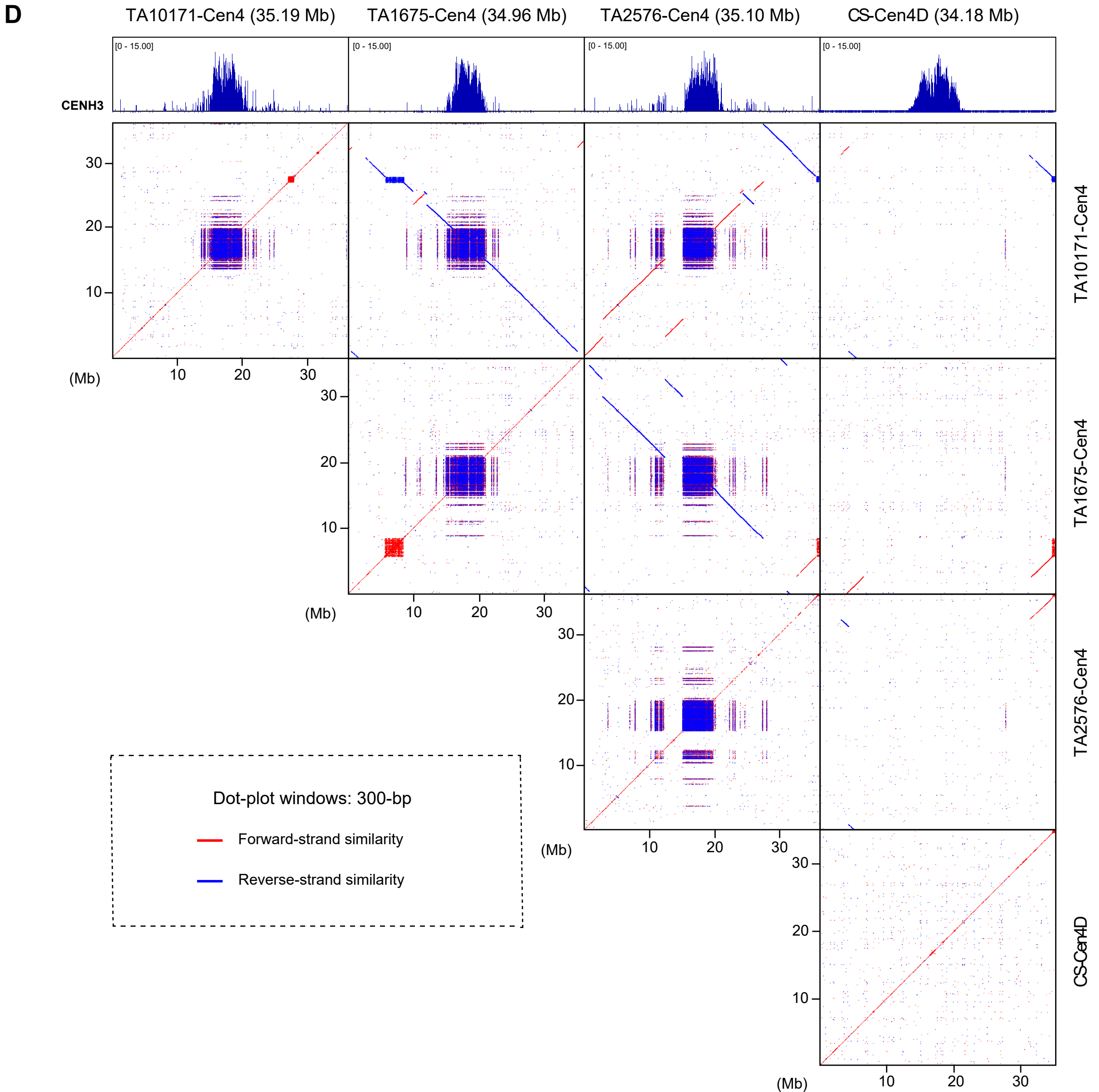
D
TA10171-Cen4 (35.19 Mb)
TA1675-Cen4 (34.96 Mb)
TA2576-Cen4 (35.10 Mb)
CS-Cen4D (34.18 Mb)
[0 - 15.00]
CENH3
TA10171-Cen4
TA1675-Cen4
TA2576-Cen4
CS-Cen4D
(Mb)
10
20
30
Dot-plot windows: 300-bp
Forward-strand similarity
Reverse-strand similarity

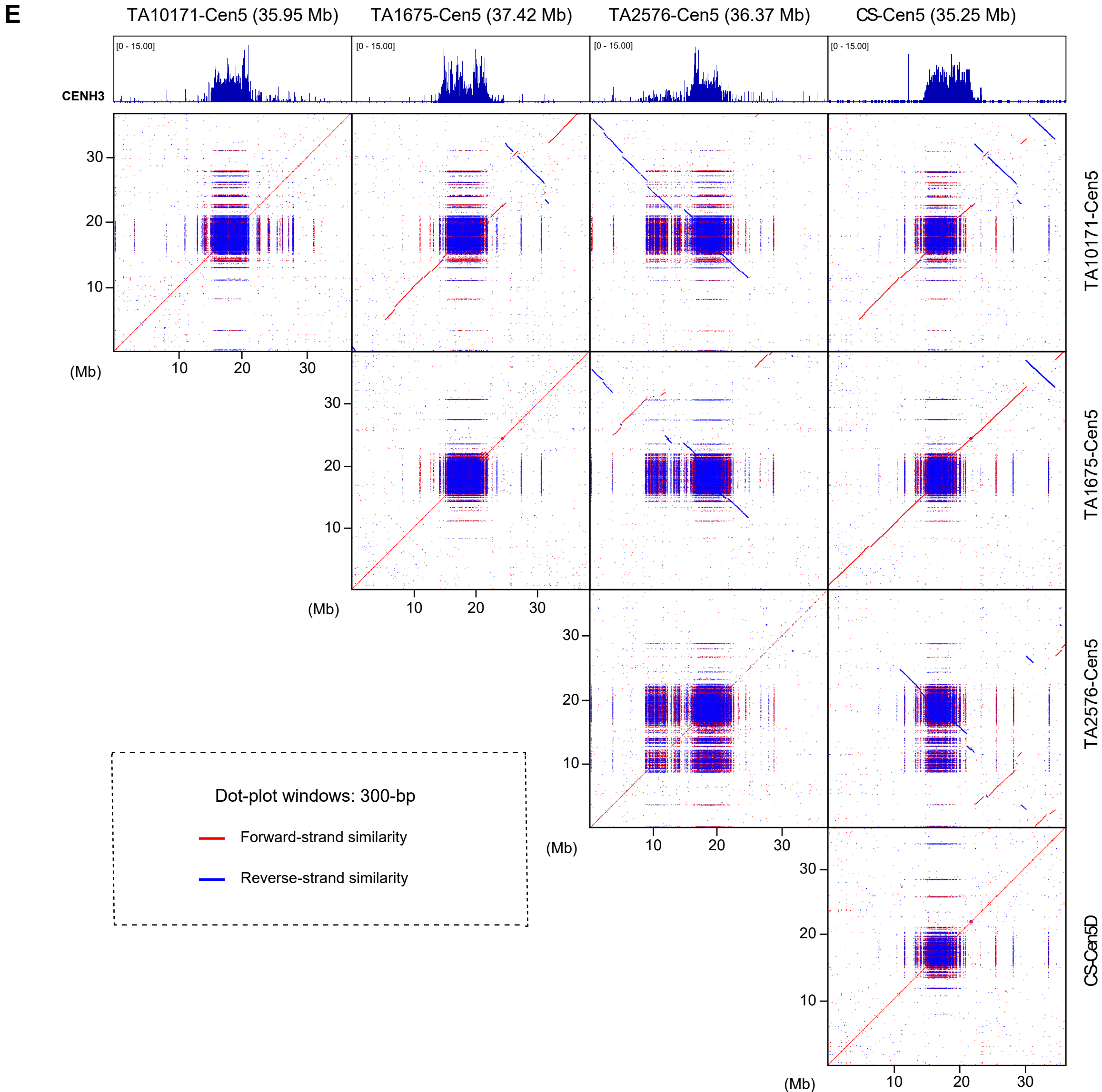

E
TA10171-Cen5 (35.95 Mb)
TA1675-Cen5 (37.42 Mb)
TA2576-Cen5 (36.37 Mb)
CS-Cen5 (35.25 Mb)
[0 - 15.00]
CENH3
TA10171-Cen5
TA1675-Cen5
TA2576-Cen5
CS-Cen5D
(Mb)
Dot-plot windows: 300-bp
Forward-strand similarity
Reverse-strand similarity

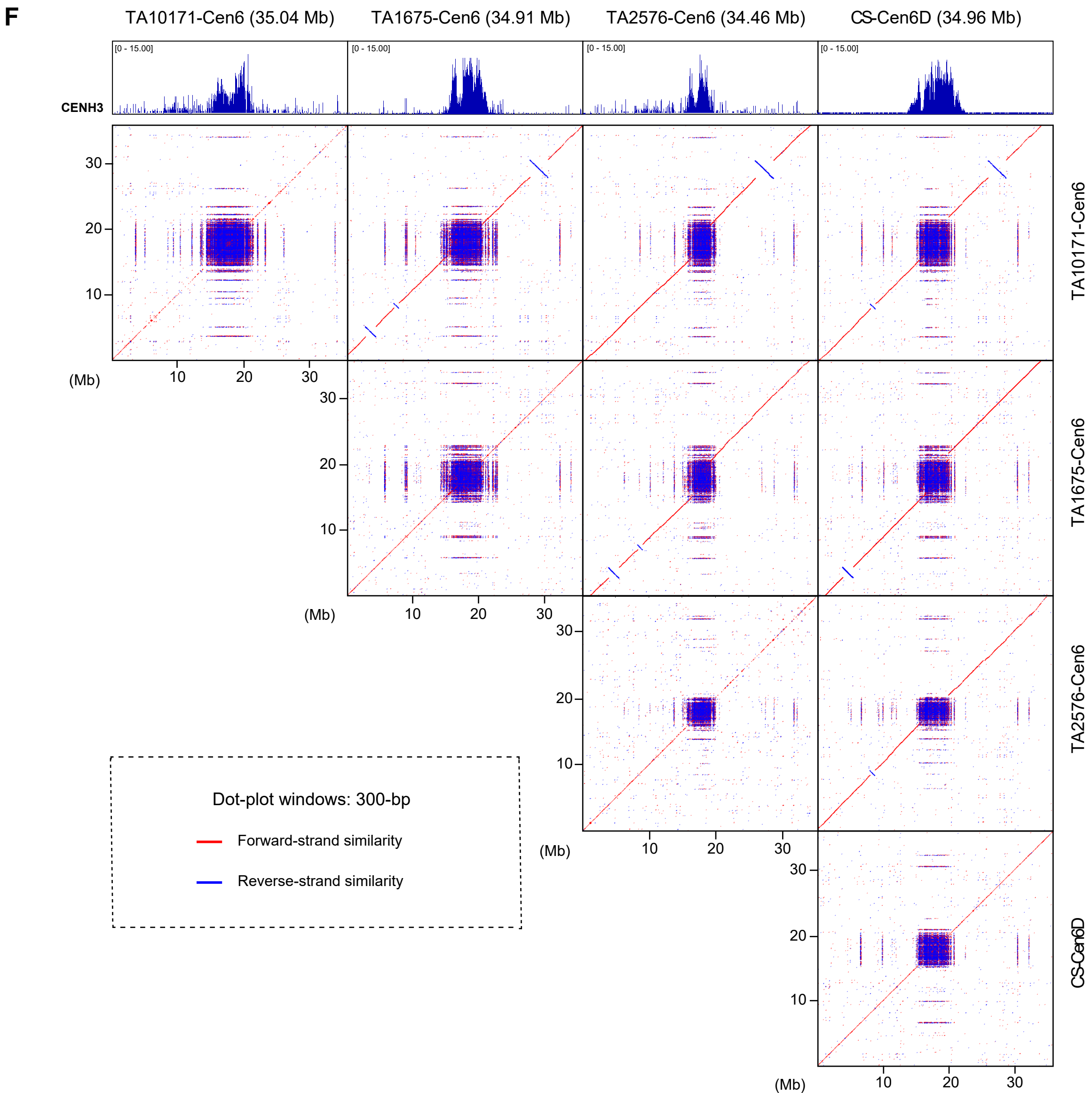
F
TA10171-Cen6 (35.04 Mb)
TA1675-Cen6 (34.91 Mb)
TA2576-Cen6 (34.46 Mb)
CS-Cen6D (34.96 Mb)
[0 - 15.00]
CENH3
TA10171-Cen6
TA1675-Cen6
TA2576-Cen6
CS-Cen6D
(Mb)
10
20
30
Dot-plot windows: 300-bp
Forward-strand similarity
Reverse-strand similarity

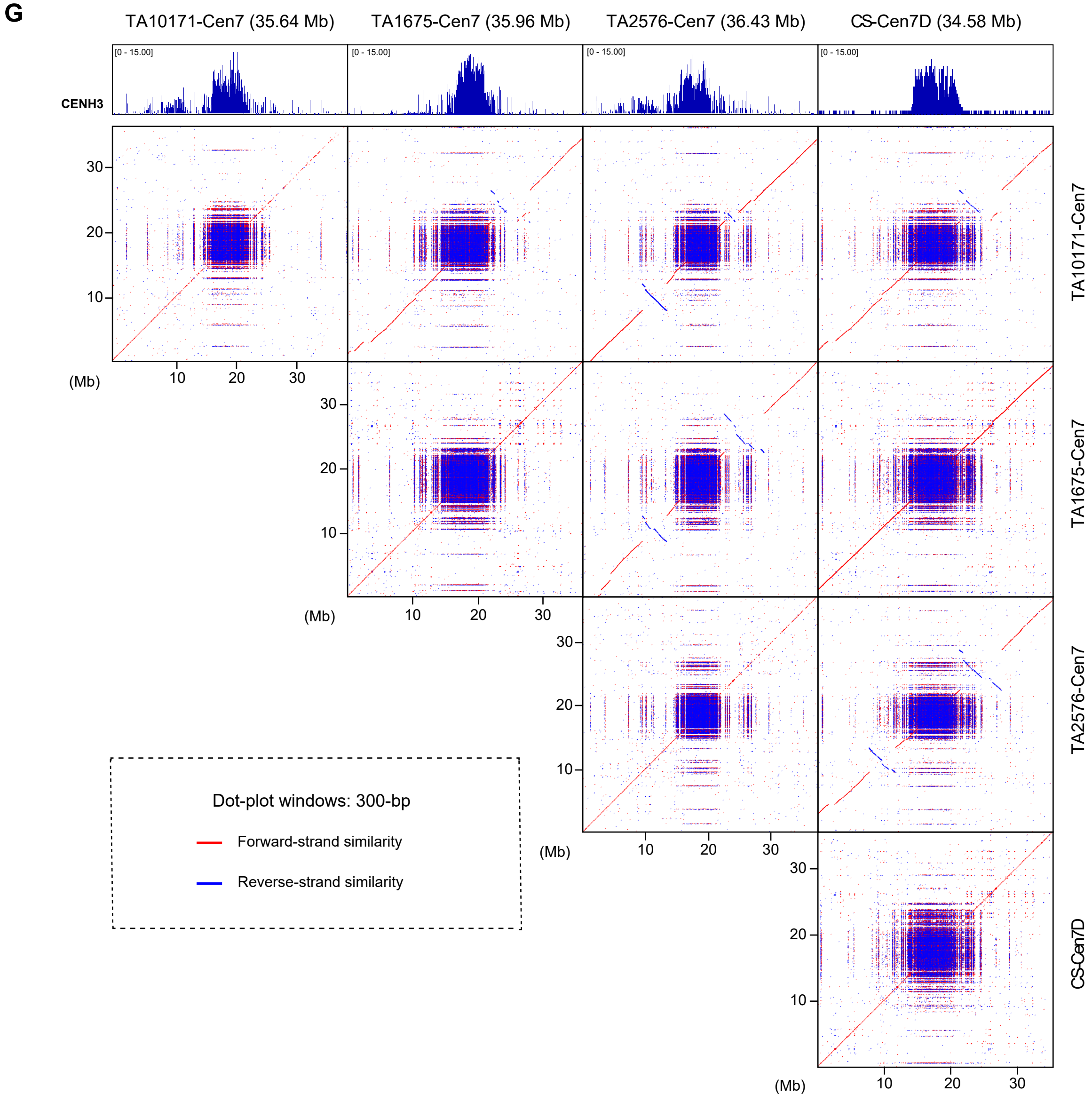
G
TA10171-Cen7 (35.64 Mb)
TA1675-Cen7 (35.96 Mb)
TA2576-Cen7 (36.43 Mb)
CS-Cen7D (34.58 Mb)
CENH3
[0 - 15.00]
TA10171-Cen7
TA1675-Cen7
TA2576-Cen7
CS-Cen7D
(Mb)
Dot-plot windows: 300-bp
Forward-strand similarity
Reverse-strand similarity

Fig. S10. Comparative analysis of D-genome centromere architecture between *Ae. tauschii* and *T. aestivum*. Note that there is little similarity between *T. aestivum*-Cen4D and *Ae. tauschii*-Cen4D. (A-G) Dot plot alignments (300-bp window) of peri/centromeric regions for chromosomes 1D–7D, comparing *Ae. tauschii* (TA10171 [L1], TA1675 and AY61 [L2], TA2576 [L3]), and *T. aestivum* (CS). Genomic coordinates (top) and CENH3 ChIP-seq coverage profiles (middle) demarcate centromere boundaries. CRWs distributions are annotated as colored bars. Forward- and reverse-strand sequence similarities are indicated in red and blue, respectively.

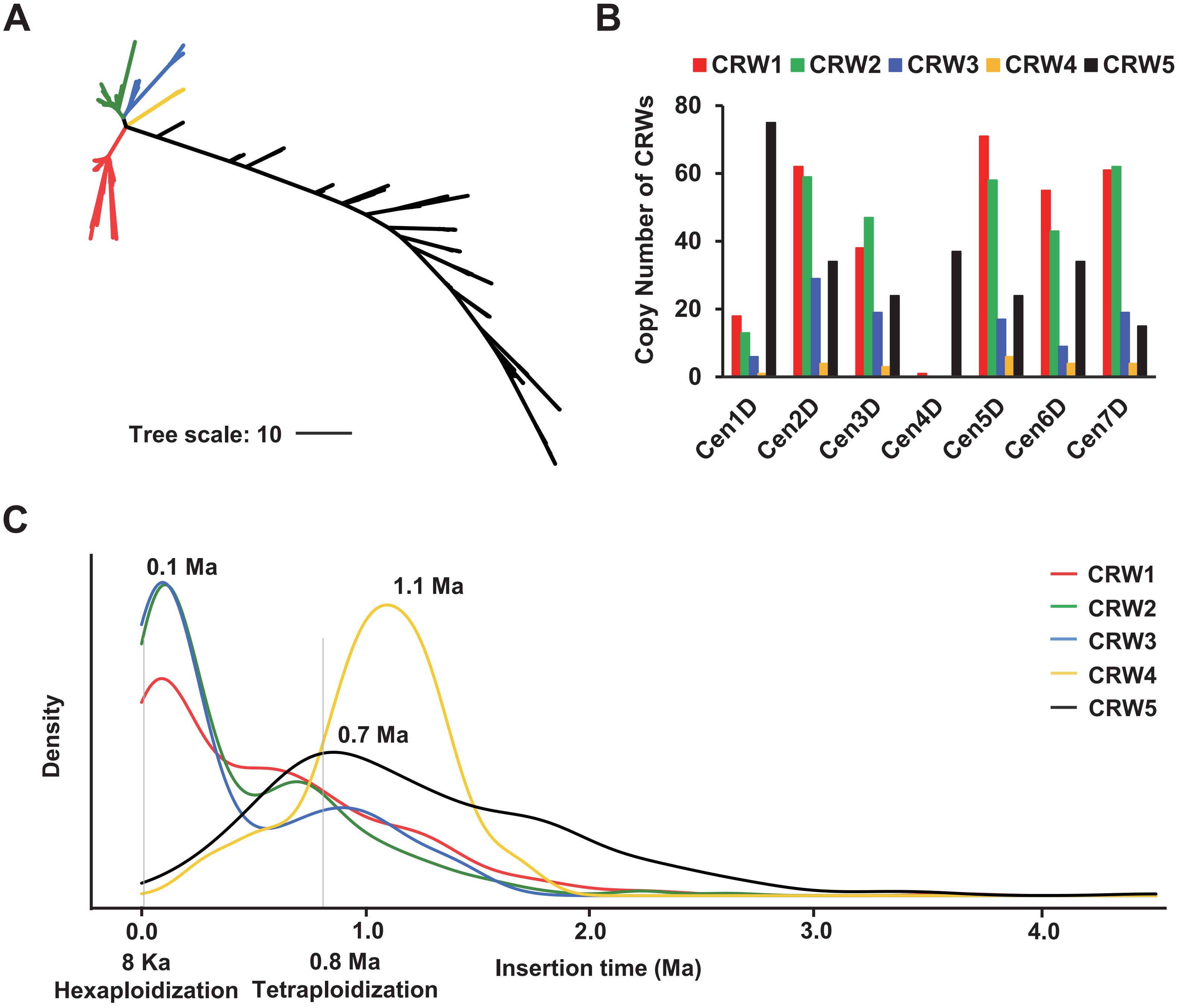
A
Tree scale: 10
B
CRW1
CRW2
CRW3
CRW4
CRW5
Copy Number of CRWs
80
60
40
20
0
Cen1D
Cen2D
Cen3D
Cen4D
Cen5D
Cen6D
Cen7D
C
0.1 Ma
1.1 Ma
0.7 Ma
CRW1
CRW2
CRW3
CRW4
CRW5
Density
0.0
1.0
2.0
3.0
4.0
8 Ka
Hexaploidization
0.8 Ma
Tetraploidization
Insertion time (Ma)

Fig. S11. Bursts of CRWs in CS-DD. (A) Phylogenetic relationship of CRWs in the DD sub-centromere of *T. aestivum*. CRW1 is red, CRW2 in green, CRW3 in blue, CRW4 in yellow, and CRW5 in black. (B) Distribution of CRWs across the seven CS-DD chromosomes. CRW1 is represented in red, CRW2 in green, CRW3 in blue, CRW4 in yellow, and CRW5 in black. (C) Density distribution of CRWs insertion time in the DD sub-centromeres of *T. aestivum*. The x-axis represents the time (Ma) of CRWs insertion. Two polyploid events are marked by dashed lines.

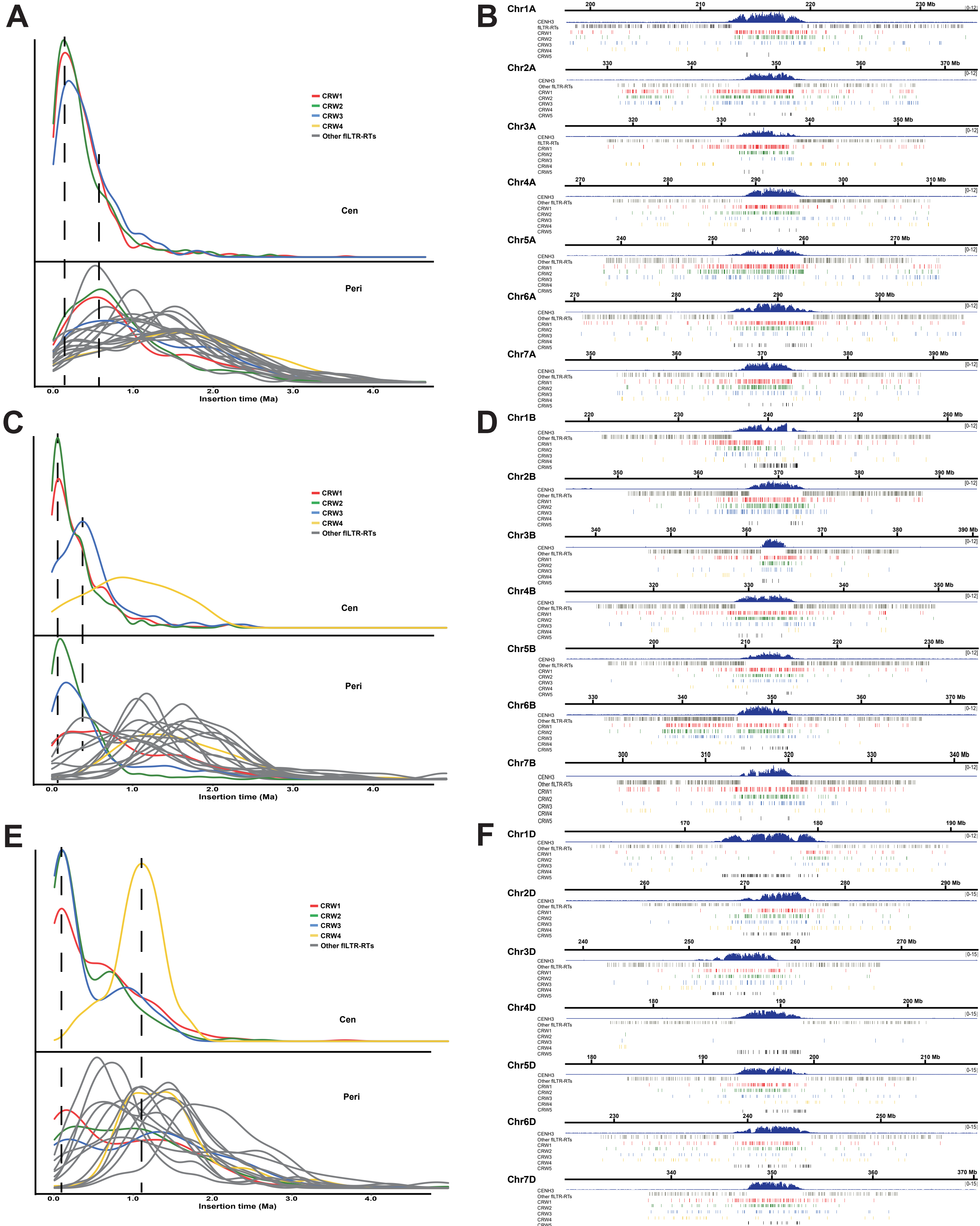
A
CRW1
CRW2
CRW3
CRW4
Other flLTR-RTs
Cen
Peri
Insertion time (Ma)
B
Chr1A
Chr2A
Chr3A
Chr4A
Chr5A
Chr6A
Chr7A
CENH3
Other flLTR-RTs
CRW5
C
D
Chr1B
Chr2B
Chr3B
Chr4B
Chr5B
Chr6B
Chr7B
E
F
Chr1D
Chr2D
Chr3D
Chr4D
Chr5D
Chr6D
Chr7D

Fig. S12. The flLTR-RTs in pericentromeres are older than those in centromeres of *T. aestivum*. (A, C, E) Kernel density estimate plots showing the insertion times of flLTR-RTs in AA (A), BB (C), DD (E) genomes of *T. aestivum*. CRW1 is shown in red, CRW2 in green, CRW3 in blue, CRW4 in yellow, and all other types of flLTR-RTs in gray. Peaks of CRWs bursts in centromeres are shown above, and those in pericentromeres are shown below. Note that no CRW4 was found in the AA subcentromere. (B, D, F**)** Distribution pattern of CRW1 (red), CRW2 (green), CRW3 (blue), CRW4 (yellow), CRW5 (black), and other flLTR-RTs (gray) in the pericentromeres of the AA (B), BB (D), DD (F) subgenomes of *T. aestivum*. From top to bottom are the homoeologous groups Chr1 to Chr7, and centromeres are located at blue peaks.
