# Supplementary material for: Distinct evolutionary trajectories of subgenomic centromeres in polyploid wheat
Source: Genome Biol. 2025 Sep 9;26:271. doi: 10.1186/s13059-025-03759-4 (PMC12418699; doi:10.1186/s13059-025-03759-4)
Supplement: Supplementary file 2 — Additional file 2. This file contains Tables S1-S2. [file 13059_2025_3759_MOESM2_ESM.pdf]

Table S1. CRWs copy numbers at DD subcentromeres in *T. aestivum*.

**Cen**	**CRW1**	**CRW2**	**CRW3**	**CRW4**	**CRW5**	**CRW5/CRWs ratio (%)**
1D	18	13	6	1	75	66
2D	62	59	29	4	34	18
3D	38	47	19	3	24	18
4D	1	0	0	0	37	97
5D	71	58	17	6	24	14
6D	55	43	9	4	34	23
7D	61	62	19	4	15	9
Total	306	282	99	22	243	

Table S2. Primers used in this study.

CRW1&2-LTR-1F	ATGCGCAAGGGGATCAAGAA
CRW1&2-LTR-1R	GGGTCCGAAACCAACCCTAA
CRW1&2-LTR-2F	ATTGTTACACCCACAGCCCC
CRW1&2-LTR-2R	GGCGGCTTCAAAGTCCTGAA
CRW3-LTR-1F	TCGGACTGCACAGACATACC
CRW3-LTR-1R	TTCGTCCACAACTCAACGCT
CRW3-LTR-2F	TCCAAGTCTCTCACGTTCTGG
CRW3-LTR-2R	TCCACAACTCAACGCTCCG
CRW4-LTR-1F	GAATCACGAAGAACACGCGG
CRW4-LTR-1R	TCCTCACGGAGAAGATCGGT
CRW4-LTR-2F	GTCTGGTCGCTGTAGCCTTC
CRW4-LTR-2R	CTCTCGAGACCGAGGTGACT
